# Hepatic Response of Magnesium-Restricted Wild Type Mice

**DOI:** 10.3390/metabo11110762

**Published:** 2021-11-06

**Authors:** Vera H. Fengler, Tanja Macheiner, Walter Goessler, Maria Ratzer, Johannes Haybaeck, Karine Sargsyan

**Affiliations:** 1 Biobank Graz, Medical University of Graz, 8036 Graz, Austria; vera.fengler@uni-graz.at; 2 Institute of Molecular Biosciences, University of Graz, 8010 Graz, Austria; 3 International Biobank and Education, Medical University of Graz, 8036 Graz, Austria; tanja.macheiner@medunigraz.at; 4 Institute of Chemistry, University of Graz, 8010 Graz, Austria; walter.goessler@uni-graz.at; 5 Institute for Biomedicine and Health Sciences, Joanneum Research, 8010 Graz, Austria; Maria.Ratzer@joanneum.at; 6 Department of Pathology, Neuropathology and Molecular Pathology, Medical University of Innsbruck, 6020 Innsbruck, Austria; Johannes.Haybaeck@i-med.ac.at; 7 Diagnostic & Research Center for Molecular BioMedicine, Institute of Pathology, Medical University of Graz, 8036 Graz, Austria

**Keywords:** magnesium-restriction, magnesium-deficiency, experimental mouse model, non-alcoholic fatty liver disease, hepatic inflammation, hepatic steatosis

## Abstract

Magnesium-deficiency is implicated in many metabolic disorders, e.g., type 2 diabetes and metabolic syndrome, representing risk factors for non-alcoholic fatty liver disease (NAFLD). This study aims to investigate the contribution of magnesium-restriction to the development of NAFLD. Magnesium-deficiency was induced in C57BL/6 mice by feeding a magnesium-deficient-diet. Metabolic markers as well as markers of inflammation and liver function were assessed. Furthermore, liver tissue was examined histopathologically and compared with specimens from high-fat-diet fed and control mice. Finally, the hepatic inflammatory response was quantified by determining hepatic IL-6, TNFα, and MCP-1. Magnesium-restriction resulted in at least a 2-fold significant reduction of serum magnesium levels compared to the high-fat-diet fed and control mice, whereas the hepatic magnesium content was decreased due to high-fat-diet feeding. No changes in metabolic markers in magnesium-restricted mice were observed, while the cholesterol content was elevated in high-fat-diet fed mice. Magnesium-restricted mice additionally featured inflammation and enlarged hepatocytes in liver histology. Furthermore, magnesium-restricted and high-fat-diet fed mice exhibited elevated hepatic TNFα levels compared to control mice. Accordingly, our data suggest that magnesium is involved in hepatic inflammatory processes and hepatocyte enlargement, key histological features of human NAFLD, and may therefore contribute to development and progression of the disease.

## 1. Introduction

Magnesium (Mg) represents the second most frequent intracellular cation in biological systems and is involved in many cellular processes, e.g., catalytical activity and specific structure of enzymes as well as regulation of ion channels [1,2]. Subclinical Mg-deficiency is thought to be quite common. The United States’ National Health and Nutrition Examination Survey from 2005 to 2006 indicates that 60% of all adults do not meet the estimated average Mg-requirement from food [3].

The necessity of Mg within the physiology of cells implicates Mg-deficiency in many metabolic disorders [4], e.g., type 2 diabetes (DMT2) [5,6,7,8,9], metabolic syndrome (MetS) [5,10,11], and hypertension [12] as well as in liver disease [13,14]. Thereby, DMT2 as well as the MetS represent risk factors for the development of non-alcoholic fatty liver disease (NAFLD) [15,16]. NAFLD is a frequent liver pathology in Western countries and clinical studies confirmed a nearly 2-fold increased mortality for NAFLD patients [17,18,19,20]. Due to caloric over-nutrition NAFLD can mature, which is characterized by hepatic steatosis and inflammation, a condition termed as non-alcoholic steatohepatitis (NASH), and can further progress to significant fibrosis and even cirrhosis. NASH affects an estimated 6% of the American United States population and leads to an annual all-cause mortality rate of 25.56 per 1000 person-years [21]. Additionally, histological features of NAFLD include hepatocellular degeneration, i.e., ballooning and Mallory Denk body formation [22,23].

Several in vitro experiments as well as in vivo studies in rats revealed Mg-deficiency influencing the insulin signaling pathway and promoting hepatic IR, which can be compensated by dietary supplementation [24,25,26,27,28]. These rats exhibited complex changes in hepatic lipid metabolism, apolipoprotein gene expression, and IR [5,24,25,29]. Another in vivo study demonstrated hepatic interleukin 6 (IL-6) and tumor necrosis factor α (TNFα) transcription as being increased in Mg-restricted mice [30]. In existing in vivo studies, mice were fed Mg-deficient diet for four days up to four weeks, which seems to be the maximum period for mice surviving complete Mg-deprivation. Accordingly, Mg-restriction led to a number of pathological responses in animal models [31,32], including systemic and unspecific inflammation [33] and as a consequence oxidative stress with increased tissue, erythrocyte, and lipoprotein peroxidation [34,35,36], reduced levels of antioxidants [37], and decreased plasma nitric oxide concentrations [38].

Clinical studies confirmed that patients with Mg-deficiency exhibit systemic inflammation [31,39] and perturbations in Mg homeostasis are related to an increased inflammatory response, mitochondrial dysfunction, and decreased antioxidant capacity [32,40]. Moreover, an epidemiological study linked low serum Mg-concentrations to insulin resistance (IR) in obese patients and patients with NASH [41]. Due to the connection between Mg-deficiency and IR and the relationship between IR and NAFLD, Patrick et al. postulated Mg-deficiency as a potential risk factor for NAFLD [42,43]. Thereby, it is thought that dietary Mg-deficiency is not the primary cause of metabolic diseases but low-grade inflammation is a major factor. Nevertheless, Mg-deficiency may enhance the inflammatory or oxidative stress induced by other factors in NAFLD. Low Mg-intake may increase chronic inflammatory stress and thereby affect the severity of pathological conditions [32]. However, data on pathophysiological changes within the liver due to Mg-restriction are limited.

We investigated if Mg-deficient nutrition has some implication on hepatic inflammatory events, contributing to the development and progression of NAFLD. In order to assess effects of Mg-restriction, male C57BL/6 wild type mice were fed with Mg-deficient-diet and deionized water for four weeks ad libitum, leading to a significant reduction of serum Mg-levels. Mg-restricted mice exhibited liver inflammation with significantly increased hepatic TNFα levels. Additionally, histological features of Mg-restricted mice include liver inflammation and enlarged hepatocytes, conditions comparable to some histological characteristics of human NAFLD. All data were compared to high-fat-diet (HF) fed mice and mice fed a normal, unrestricted chow [44].

## 2. Results

### 2.1. Mg-Concentration in Sera and Livers of Mg-Restricted and HF-Diet Fed Mice

**Figure 1:**
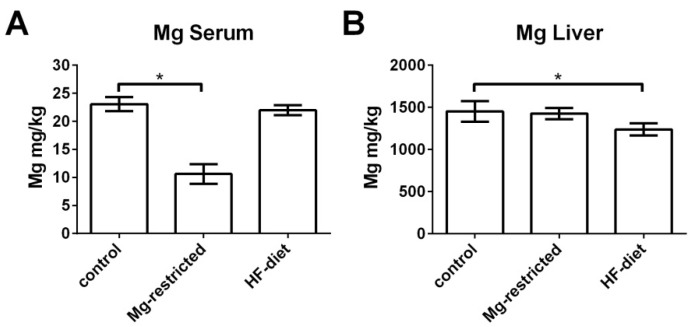
Serum and liver Mg-concentrations in control, Mg-restricted, and HF-diet fed mice. Mg-content was measured with ICPMS resulting in (**A**) significant reduced serum Mg-concentrations in Mg-restricted mice compared to control mice. (**B**) Hepatic Mg-content is not reduced in Mg-restricted mice, while HF-diet feeding results in significantly lower liver Mg-content as compared to control mice. Depicted are means with standard deviations and *p* values of < 0.05 are marked by an * (one-way ANOVA followed by Dunnett’s multiple comparisons test).

In order to investigate the impact of four weeks Mg-restriction on systemic and hepatic Mg-content, Mg-concentration in serum and liver were determined by ICP-MS. Serum Mg-concentration descended approximately 2-fold due to Mg-restriction (*p* < 0.05), which was reported previously [45]. HF-diet feeding caused no alterations in serum Mg-concentration (Figure 1A). In contrast, hepatic Mg-content was not reduced in Mg-restricted mice, while in HF-diet fed mice a significant lower hepatic Mg-concentration as compared to control mice was observed (*p* < 0.05) (Figure 1B).

### 2.2. Body Parameters of Mg-Restricted and HF-Diet Fed Mice

**Figure 2:**
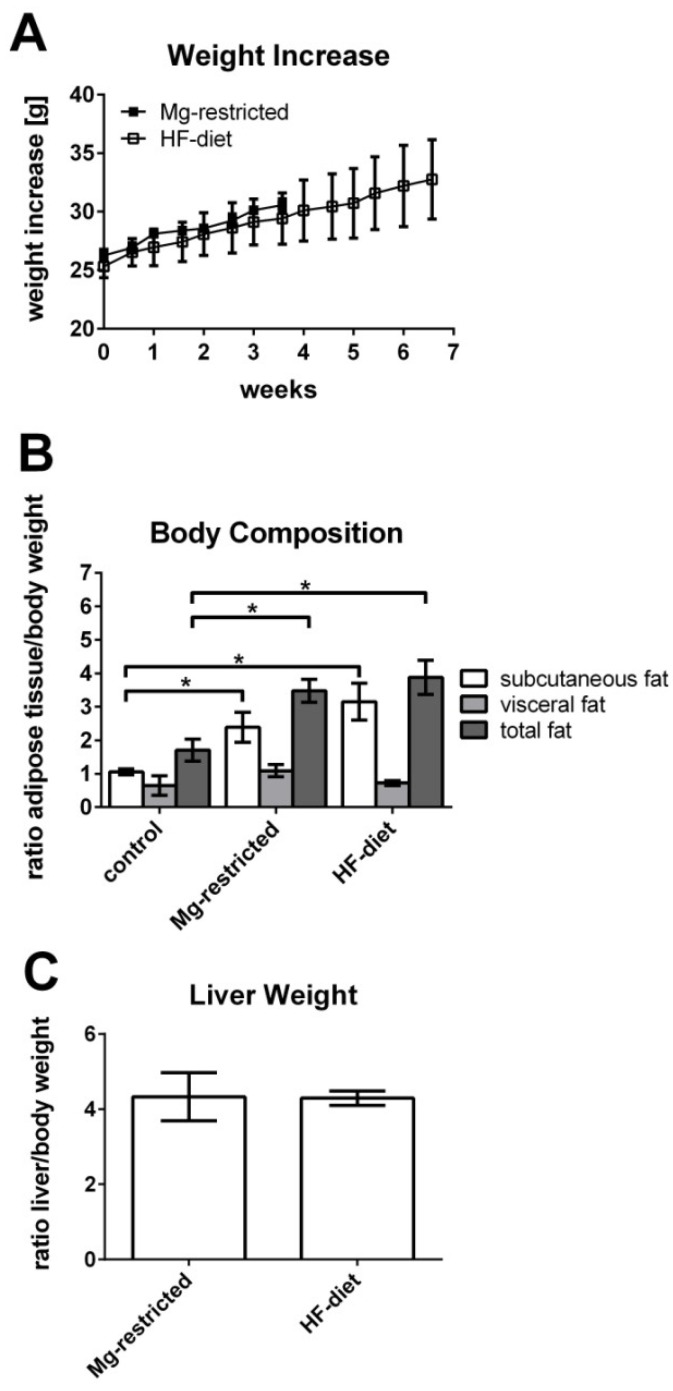
Weight increase and body composition of Mg-restricted and HF-diet fed mice. (**A**) Weight of mice was monitored twice a week and no statistically significant differences in weight increase are observed between the two different dietary treatments. (**B**) Subcutaneous and visceral fat pads of all mice were dissected, weighed, and respective adipose tissue to bodyweight ratios were calculated, showing significant differences between Mg-restricted and HF-diet fed mice compared to control mice. Shown are means with standard deviations and significant differences are marked with an * (*p* < 0.05 by one-way ANOVA followed by Dunnett’s multiple comparisons test). (**C**) Livers of dietary treated mice were dissected, weighted, and respective liver to bodyweight ratios were calculated, showing no significant differences between Mg-restricted and HF-diet fed mice.

To monitor body weight increase of Mg-restricted mice, animals were weighed twice a week, body weight increase was calculated, and compared with HF-diet fed mice. Mg-restricted and HF-diet fed mice showed no statistically significant differences in weight increase (Figure 2A). Withal, weight increase of both dietary groups was comparable to weight increase of adult C57BL/6 mice of the same age, fed with standard chow as it is reported in literature [30,44,46]. Additionally, body composition was observed at the end of the experiment by dissecting and weighing subcutaneous and visceral adipose tissue to further calculate subcutaneous adipose tissue, visceral adipose tissue, and total adipose tissue to body weight ratios. In contrast to weight increase, body composition was influenced by the two different dietary treatments. Total fat was increased in both Mg-restricted as well as HF-diet fed mice compared to control mice (*p* < 0.05). Additionally, subcutaneous fat was elevated significantly in both dietary groups compared to control mice (*p* < 0.05), while visceral fat was not affected by dietary treatments (Figure 2B). Liver weight did not change due to different dietary treatments (Figure 2C).

### 2.3. Influence of Mg-Restriction and HF-Diet Feeding on Systemic Metabolic Parameters and Liver Markers

**Figure 3:**
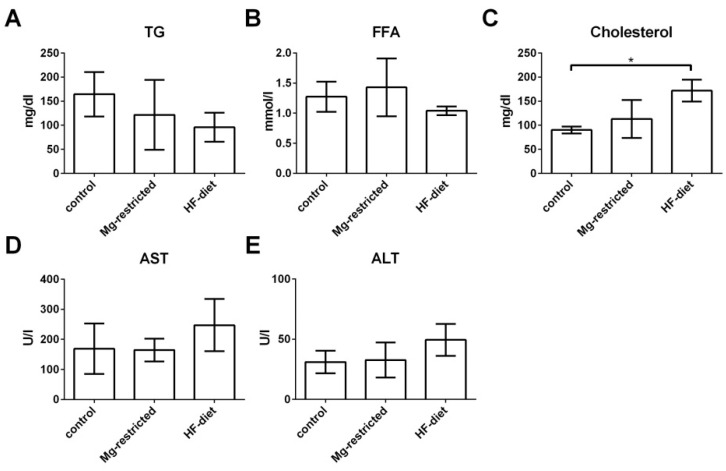
Influence of Mg-restriction and HF-diet feeding on metabolic serum parameters and serum markers of liver function. Levels of TG, FFA, total cholesterol, and transaminases AST and ALT determined out of serum, resulting in no significant changes in (**A**) TG and (**B**) FFA, while (**C**) total cholesterol levels are significantly increased in HF-diet fed mice. Serum levels of (**D**) AST and (**E**) ALT are not altered due to dietary treatments. Given are means with standard deviations and relevant significant differences are marked with an * (*p* values of < 0.05 by one-way ANOVA followed by Dunnett’s multiple comparisons test).

Systemic metabolic changes and disturbances in liver function were assessed by determining serum concentrations of triglycerides (TG), free fatty acids (FFA), cholesterol, aspartate aminotransferase (AST), and alanine aminotransferase (ALT). Mg-restriction as well as HF-diet feeding resulted in no significant reduction of serum TG and no accumulation of FFA in sera of the respective mice (Figure 3A,B). In contrast, total cholesterol was elevated in HF-diet fed mice compared to control mice (*p* < 0.05) (Figure 3C). AST as well as ALT levels were elevated due to HF-diet feeding. However, these increases did not reach statistical significance (Figure 3D,E).

### 2.4. Determination of Hepatic Steatosis, Ballooning, and Inflammation in Mg-Deficient and HF-Diet Fed Mice

**Figure 4:**
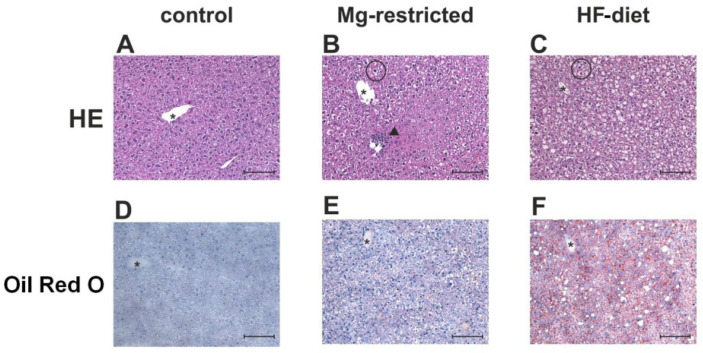
HE and oil red O stained liver sections of control, Mg-restricted, and HF-diet fed mice. To obtain an overall picture of liver histology, all liver sections of (**A**) control, (**B**) Mg-restricted, and (**C**) HF-diet fed mice are HE stained. Oil red O staining was performed to illustrate lipid accumulation in livers of (**D**) control, (**E**) Mg-restricted, and (**F**) HF-diet fed mice. Depicted are representative excerpts of the respective group (magnification 200), in which asterisks mark a central vein, black triangles heads mark inflammatory cell infiltration, and circles indicate enlarged hepatocytes, probably representing ballooning. The length of the scale bar in each picture is 100 µm.

To investigate the influence of Mg-restriction in liver more specifically, liver sections of control, Mg-restricted, and HF-diet fed mice were stained with hematoxylin/eosin (HE) and oil red O (Figure 4). Livers of control mice appeared histologically normal, with no signs of lipid accumulation, inflammation, and ballooning (Figure 4A,D). In contrast, livers of Mg-restricted mice exhibited infiltrated inflammatory cells as well as enlarged hepatocytes, possibly representing an initial stage of ballooning (Figure 4B). Furthermore, oil red O stained liver slides of Mg-restricted mice displayed evidence of accumulating lipids, featured by small aggregates surrounding hepatocytes (Figure 4E). Livers of HF-diet fed mice showed steatosis and enlarged hepatocytes, resembling ballooning (Figure 4C,F). Nevertheless, inflammatory cells were minimal in these livers compared to Mg-restricted mice (Figure 4C).

Additionally, all liver sections were histopathologically examined and scored as described in Section 4. The scoring of liver sections from Mg-restricted mice resulted in a numerically higher inflammation, ballooning, and sum score (Table 1). Steatosis, ballooning, and the respective sum score of HF-diet fed mice were statistically significantly elevated compared to control mice, while inflammation was not increased (*p* < 0.05) (Table 1).

### 2.5. Hepatic Inflammatory Response to Mg-Restriction and HF-Diet Feeding

**Figure 5:**
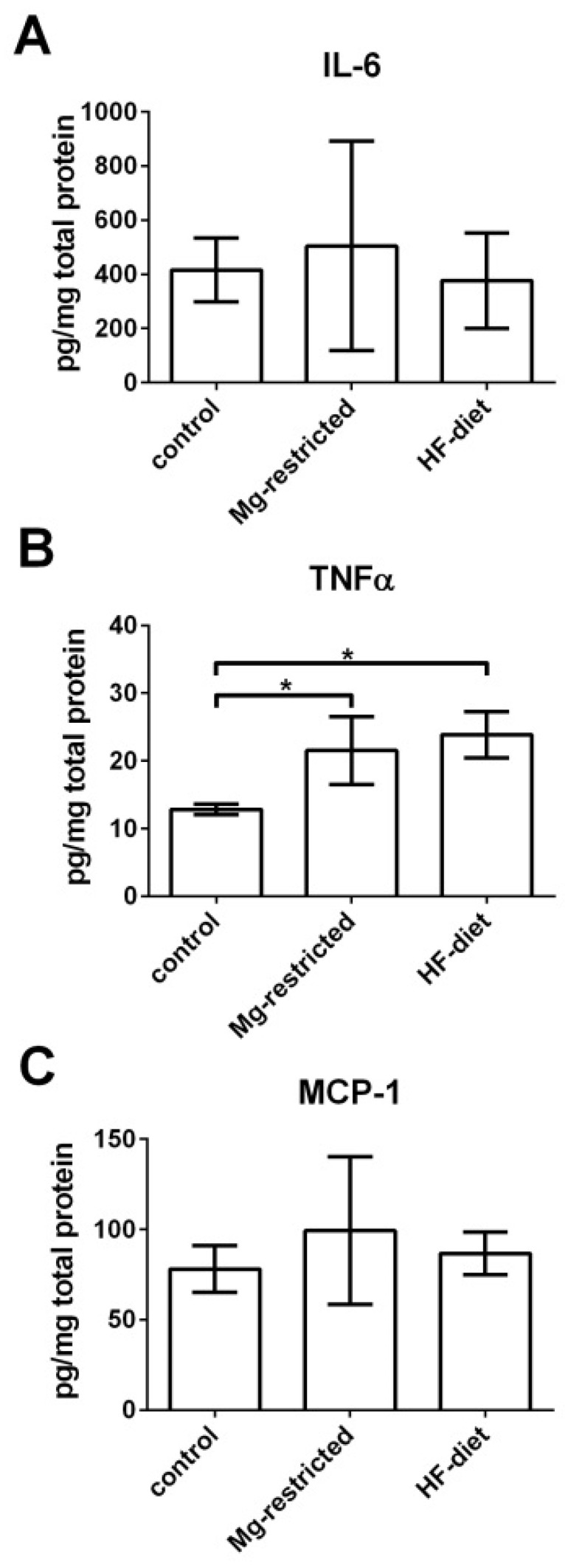
Influence of Mg-restriction and HF-diet feeding on hepatic inflammation. Levels of inflammation markers IL-6, TNFα, and MCP-1 were measured in liver homogenates, resulting in no significant alterations in (**A**) IL-6 and (**C**) MCP-1, while (**B**) TNFα levels are significantly increased in Mg-restricted and HF-diet fed mice compared to control mice. Shown are means with standard deviations and relevant significant differences are marked with an * (*p* values of < 0.05 by one-way ANOVA followed by Dunnett’s multiple comparisons test).

For quantification of the hepatic inflammatory response of Mg-restricted and HF-diet fed mice, IL-6, TNFα, and monocyte chemoattractant protein-1 (MCP-1) were determined from liver homogenates and calculated as pg per mg total protein. A comparison of IL-6 levels between Mg-restricted, control, and HF-diet fed mice was not possible due to the high standard deviation in the Mg-restricted group (Figure 5A). TNFα, an enhancing factor of fibrosis in NASH [47,48], was significantly increased upon to Mg-restriction and HF-diet feeding (*p* < 0.05) (Figure 5B). MCP-1 levels instead were not elevated in livers of Mg-restricted and HF-fed mice compared to control mice (Figure 5C).

## 3. Discussion

Administration of Mg-deficient-diet to mice for four weeks leads to an approximately 2-fold decrease of serum Mg-concentration, representing a systemic Mg-deficiency in our model. Interestingly, this systemic Mg-deficiency was not sufficient to reduce hepatic Mg-content. This is in contrast to what was observed in rats receiving a Mg-deficient-diet for 2 weeks, exhibiting a significant decrease in hepatic Mg-content. Whether four weeks of Mg-restriction in mice is insufficient to induce hepatic Mg-deficiency or whether physiological mechanisms specifically maintain hepatic Mg-content in mice during systemic Mg-deficiency requires further elucidation.

In our study, Mg-restriction does not affect body weight increase of mice fed a Mg-deficient diet for four weeks. Body weight gain of Mg-restricted rodents is discussed controversially in the literature. Some studies did not observe any influence of Mg-restriction on body weight [26,28], while others showed retarded body weight gain due to Mg-restriction [30,49]. Nevertheless, it was observed that the offspring of Mg-restricted rat dams exhibited adiposity [26]. Here, a change in body composition is observed due to Mg-restriction, similar to that induced by HF-diet feeding. Moreover, visceral adipose tissue is increased as a response to Mg-restriction, which is a feature of the MetS [15] and NAFLD [50] in humans. Serum TG levels of Mg-restricted mice are slightly decreased. Mg-restriction caused also a slight increase of serum FFA and total cholesterol concentrations. Although all these differences reached no statistical significance, they might indicate metabolic disturbances also affecting liver metabolism. These features of the MetS and NAFLD have been recapitulated in rodent models of Mg-restriction [29,49,51]. Other features of the MS and NAFLD have also been shown in rodent models of Mg-restriction, e.g., elevated serum and hepatic TG levels [28,37] as well as an increased serum cholesterol concentration [35]. In this study, serum TG levels of Mg-restricted mice are slightly decreased, which may point towards an elevated lipoprotein lipase activation and resulting TG clearance, also observed in some mouse models of NAFLD [29,38]. Mg-restriction in this study caused also a slight increase of serum FFA and total cholesterol concentrations. Although all these differences reached no statistical significance, they might indicate metabolic disturbances also affecting liver metabolism. An in vitro study revealed that reduced extracellular and cellular Mg-concentrations interfere with the accumulation of glucose in hepatocytes and their ability to metabolize glucose accurately. Thereby, glucose metabolism and insulin resistance are altered [22]. Apart from hepatic glucose metabolism, Mg-restriction influences cholesterol composition, resulting in elevated VLDL and LDL cholesterol and decreased HDL cholesterol, by reducing the activity of lecithin-cholesterol acyltransferase [40].

We observed enlarged hepatocytes and small aggregates of accumulating lipids surrounding hepatocytes in livers of Mg-restricted mice. The enlarged hepatocytes might represent initial stages of ballooning cell degeneration, occurring during human NAFLD. Most prominent in liver histology was the infiltration of inflammatory cells during Mg-restriction in mice. Inflammatory foci are observed near the portal triade and are absent in HF-diet fed mice. A similar liver histology was previously found in Mg-restricted rats, with inflammatory cells located around the portal triade and enlarged vacuoles in hepatocytes, which might consist of lipids [52]. These initial features of NAFLD in livers of Mg-restricted rodents indicate a role for Mg in setting conditions making the liver susceptible for the development of NAFLD. If a more pronounced hepatic injury can be induced by prolonging the period of Mg-restriction or by combining Mg-restriction with HF-diet, feeding has to be further investigated.

In our Mg-restricted mouse model, hepatic TNFα, a key factor in the development of NASH-associated fibrosis [53], is significantly increased. This might be due to the elevated presence of mast cells, secreting TNFα and other molecules enhancing fibrosis [52]. Hepatic IL-6 levels were only slightly decreased in Mg-restricted mice, but this may also indicate a hepatic inflammatory response to Mg-restriction [47,48,53], finally leading to oxidative stress as well as lipid metabolism disorders [31,39]. IL-6 levels showed no significant difference between Mg-restricted, control, and HF-diet fed mice probably due to the high standard deviation in the Mg-restricted group. Regardless, decreased hepatic IL-6 indicates NAFLD-like liver injury [47,48]. In rodent models of NAFLD, inflammation is initiated by MCP-1 [46], but in our study, MCP-1 levels were not elevated in the livers of Mg-restricted and HF-fed mice compared to control mice. The observed changes may indicate increased oxidative stress in the livers of Mg-restricted mice. Therefore, further studies investigating the oxidative situation by measuring malondialdehyde levels or the ratio of glutathione to glutathione disulfide in sera as well as in the liver of the respective treated mice would be of high interest.

As far as we know, no studies in NAFLD or NASH patients, investigating the influence of Mg-levels on liver enzymes AST and ALT, as well as liver histology in humans exist. However, evidence of an influence of Mg-deficiency on NAFLD via IR, suggest an evaluation of Mg-status in NAFLD [43]. With this study, we showed that Mg-restriction in mice induces hepatic inflammation and NAFLD-associated liver injury. Accordingly, Mg-deficiency may contribute to the development and progression of NAFLD by modulation of hepatic inflammatory processes and hepatocyte degeneration, not only in mice but also in humans.

## 4. Materials and Methods

### 4.1. Animal Model and Diets

Mice were used in this study in strict accordance to the Animal Welfare Policy of the Medical University of Graz and housing was conducted with food and water ad libitum. The animal protocol (BMWF-66.010/0081-II/3b/2012) has been approved by the Austrian Federal Ministry of Science, Research Ref. II/3b and monitored in accordance with the Animal Welfare Committee of the Medical University of Graz.

The 10 week old male C57BL/6N (C57BL/6) mice (Charles River Laboratories, Sulzfeld, Germany) were weight matched, divided into three groups, and group-housed in individually ventilated cages with a 12 h light and dark cycle. After the acclimatization period, the first group (*n* = 5) received a Mg-deficient-diet (ssniff^®^ EF R/M Magnesium deficient: crude fat 4.2%, Mg < 0.02%) and deionized water for 4 weeks. The second group (*n* = 5) was fed a HF-diet (liquid Lieber DeCarli: 46.23% fat: 28.17% corn oil, 16.49% olive oil, 1.57% safflower oil, Mg 0.13%) (Ssniff, Soest, Germany) for 7 weeks. The third group (*n* = 5) served as control group and was sacrificed immediately after feeding a normal chow (ssniff^®^ R/M-H: crude fat 3.3%, Mg 0.22%). Experimental data on HF-diet fed and control mice were recently reported [44]. At the end of each experiment, mice were anesthetized by isoflurane inhalation and decapitated. Blood was collected and further processed to serum by coagulation for 30 min at room temperature (RT) and centrifugation (5000 g, 15 min, RT). The liver was weighed, snap frozen, and stored in liquid nitrogen as well as fixed in 10% formalin. Weight increase of all mice was monitored twice a week and at the end of each experiment subcutaneous and visceral adipose tissue was weighed and subcutaneous adipose tissue, visceral adipose tissue, and total adipose tissue to body weight ratios were calculated.

### 4.2. Mg-Concentration in Sera and Livers

Fresh liver samples (90–130 g) were dried at 60 °C to a constant mass (32–40 mg). Dried samples were weighed to 0.1 mg and mineralized with nitric acid in a microwave-heated autoclave (UltraCLAVE IV; MLS Mikrowellen-Labor Systeme GmbH, Leutkirch, Germany). The concentration of Mg in the dried liver tissue was determined with inductively coupled plasma mass spectrometry (ICPMS) (Agilent 7500ce; Agilent Technologies, Waldbronn, Germany) at a mass-to-charge ratio of m/z = 24. Accuracy of the determined concentrations was confirmed with the standard reference material “bovine muscle” (RM 8414; NIST). Mg in the sera was also determined with ICPMS after acid digestion.

### 4.3. Biochemical Serum Parameters

Determination of serum levels of AST, ALT, TG, and cholesterol were measured with kits used for clinical chemistry determinations (Roche Diagnostics, Basel, Switzerland) and were performed according to the manufacturer’s instructions with an auto-analyzer. For the determination of FFA, 10 µL of each sample were mixed with 150 µL reagent 1 and incubated for 10 min at RT. A total of 75 µL of reagent 2 were added and incubated for additional 10 min at RT (Wako Chemicals GmbH, Osaka, Japan). Absorption was measured at 546 nm. The level of sensitivity for this measurement is 0.01 mmol/L and the range of linearity is 0.01–4.00 mmol/L.

### 4.4. Histological Features

Formalin fixed liver specimens were embedded in paraffin and sectioned at 3 μm. Slices were stained for HE and microscopic examination was conducted by an experienced, board certified pathologist (J.H.) in a blinded manner. Thereby hepatic steatosis and inflammation were quantitatively assessed and scored as follows. All samples with at least 5% steatosis covering the liver parenchyma were diagnosed as steatosis. In doing so, steatosis was rated from 0 to 3, in which <5% means 0; 5—33% 1 (mild steatosis); 34—66% 2 (moderate steatosis); >67% 3 (marked steatosis). Furthermore, the additional presence of hepatocellular ballooning (from 0 to 2) and lobular inflammatory infiltrates (no foci: 0; 2 foci per 200× field: 1; > 2 foci per 200× field: 2) were scored.

Additionally, snap frozen liver specimens were sectioned at 6 μm and stained for oil red O to visualize hepatocytic lipid content.

### 4.5. Hepatic and Serum Inflammation Parameters

Liver specimens were homogenized by using a MagNA Lyser Instrument in combination with MagNA Lyser Green Beads (Roche, Basel, Switzerland). Supernatants of liver homogenates were centrifuged twice (16,000 g, 10 min at 4 °C) and afterwards total protein concentration was measured with a DC Protein Assay Kit (Bio-Rad, Hercules, CA, USA). IL-6, TNFα, and MCP-1 content was determined with a ProcartaPlexTM Immunoassay (eBioscience, San Diego, CA, USA) by using 25 µL of diluted supernatants of liver homogenates (10 mg/mL total protein concentration).

### 4.6. Statistical Analyses

For data analyses, one-way ANOVA followed by Dunnett’s multiple comparisons test was used and results are expressed as means with standard deviations, if not stated otherwise. Differences were considered significant for *p* values of < 0.05.

## Figures and Tables

**Table 1:** Histopathological feature scoring of steatosis, ballooning, and inflammation in livers of control, Mg-restricted, and HF-diet fed mice, expressed as medians and interquartile ranges.

	Control	Mg-Restricted	Hf-Diet
Steatosis	0.0 ± 0.0	0.0 ± 0.0	0.6 * ± 0.5
Lobular inflammation	0.4 ± 0.9	0.8 ± 0.4	0.2 ± 0.4
Ballooning	0.0 ± 0.0	0.8 * ± 0.4	1.8 * ± 0.4
Sum	0.4 ± 0.9	1.6 * ± 0.5	2.6 * ± 0.5

* Differences of dietary treatment group compared to control mice were considered significant for *p* values of < 0.05 by one-way ANOVA followed by Dunnett’s multiple comparisons test.

## Data Availability

Data available within the article.

## Abbreviations

NAFLD	non-alcoholic fatty liver disease
Mg	Magnesium
DMT2	type 2 diabetes
MetS	metabolic syndrome
NASH	non-alcoholic steatohepatitis
IR	insulin resistance
IL-6	interleukin 6
TNFα	tumor necrosis factor α
HF	high-fat
TG	triglycerides
FFA	free fatty acids
AST	aspartate aminotransferase
ALT	alanine aminotransferase
HE	hematoxylin/ eosin
MCP-1	monocyte chemoattractant protein-1
RT	room temperature
ICPMS	inductively coupled plasma mass spectrometry
